# Patient Selection for Mitral Transcatheter Edge-to-Edge Repair

**DOI:** 10.14797/mdcvj.1199

**Published:** 2023-05-16

**Authors:** Habib Layoun, Serge C. Harb, Amar Krishnaswamy, Rhonda Miyasaka, James Yun, Samir R. Kapadia

**Affiliations:** 1 Cleveland Clinic, Cleveland, Ohio, US

**Keywords:** transcatheter, mitral valve repair, mitral regurgitation, patient selection

## Abstract

Mitral regurgitation (MR) is one of the most common valvular heart diseases, with many patients remaining non-suitable for surgical interventions. Transcatheter edge-to-edge repair (TEER) is a rapidly evolving procedure that allows safe and effective reduction of MR in high-risk patients. However, adequate patient selection through clinical assessment and imaging modalities remains a key factor for procedural success. In the following review, we highlight recent developments in TEER technologies that are expanding the target population and currently available imaging modalities that allow detailed evaluation of the mitral valve and surrounding structures for optimal patient selection.

## Introduction

The mitral valve is a multidimensional apparatus with complex interactions among its different components. Anatomic and/or functional disturbances within the apparatus can lead to mitral regurgitation (MR),^1^ the most common valvular heart disease.^2^ Early surgical intervention in patients with MR is important and can prevent the development of heart failure.^1,3^ However, many patients are at high risk for surgical interventions given their advanced age and multiple comorbidities.^4^

Transcatheter edge-to-edge repair (TEER) is one of many recently developed percutaneous interventions aimed at treating MR patients with high surgical risk. Currently, TEER is a Class IIa recommendation for select patients with severe MR of either primary (PMR) or secondary (SMR) etiology and high surgical risk.^5^ Nevertheless, accurate patient selection for patients undergoing TEER is essential for procedural success and safety. In this review, we discuss the current clinical and imaging criteria for optimal patient selection.

## TEER Procedural Overview

In the TEER procedure, a portion of the anterior and posterior mitral valve leaflets are fused together, akin to the “Alfieri Stitch.” The MitraClip device (Abbott Vascular), which received US Food and Drug Administration (FDA) approval for treating PMR in 2014 and SMR in 2018, is currently the most widely used device for TEER. The clip delivery system is comprised of a 24F delivery sheath that is advanced to the left atrium via the femoral vein and through which the guiding catheter is advanced with the device on its tip.^6^ Manipulation of the clip to achieve a position perpendicular to the coaptation line at the area of leaflet pathology is achieved using iterative movements of the delivery sheath and the guiding catheter.

The third generation of the MitraClip device achieved even better MR reduction than in the EVEREST and COAPT trials.^7^ Currently, the most recent generation, the MitraClip G4, is under investigation in the EXPAND G4 trial with promising preliminary results. The G4 device is available in four sizes with combinations of two different arm lengths (12 mm for XT/XTW and 9 mm for NT/NTW) and widths (4 mm for NT/XT and 6 mm for NTW/XTW).^8^ Guidance throughout the procedure is generally achieved using transesophageal echocardiography (TEE). In the contemporary era, 3-dimensional (3D) TEE with real-time multiplanar reconstruction has demonstrated great potential in guiding implantation of the device, given its ability to simultaneously display key on-axis 2D views along with the 3D volumetric representation for optimal visualization.^9^

Another TEER device, the Pascal system (Edwards Lifesciences Corp.) recently received FDA approval for treatment of PMR after showing promising outcomes and non-inferiority to the MitraClip device in the Edwards PASCAL Transcatheter Valve Repair System Pivotal Clinical Trial (CLASP IID) trial.^10^ While early study also has shown efficacy in patients with functional mitral regurgitation (FMR), the Edwards PASCAL Transcatheter Valve Repair System Pivotal Clinical Trial (CLASP IIF) is currently ongoing in the United States. The Pascal device has two sizes, the original and the narrower Ace implant (10-mm versus 6-mm paddles), with a similar delivery system as the MitraClip system. In distinction from the MitraClip device, the Pascal device has a central spacer that fills the regurgitating orifice.

## TEER in Primary MR

Primary MR reflects a pathologic change in the mitral valve apparatus itself. Subgroups of PMR could be grouped by Carpentier classification as leaflet damage and perforation, excessive leaflet motion (prolapse or flail), and leaflet restriction (rheumatic heart disease).^11^ In the Endovascular Edge-to-Edge Repair Study (EVEREST II) trial, patients with PMR were included and demonstrated non-inferiority of the MitraClip procedure to surgical intervention.^12^ Major inclusion criteria included severe MR with an ejection fraction of 25% to 60%, left ventricular end-systolic diameter ≤ 55 mm, and a regurgitant jet caused by malcoaptation.^13^ However, multiple echocardiographic exclusion criteria were implemented (ie, orifice area < 4.0 cm^2^, severe mitral annular calcification, and evidence of intracardiac mass), which highlights the importance of accurate preprocedural assessment of the mitral valve using advanced imaging technologies. The currently available device, as well as advances in imaging technology, have called into question the EVEREST anatomic criteria first set forth more than a decade ago.

## TEER in Secondary MR

Secondary MR, as the name implies, is the result of left-sided chamber enlargement/dysfunction. The most frequent type is ventricular SMR, which is due to ventricular dilation causing mitral valve annulus dilation and apical tethering of the mitral leaflets.^14^ Guideline-directed medical therapy (GDMT) remains the first recommendation for patients with SMR,^5^ with a goal to improve cardiac function and remodeling, resulting in reduction of SMR. Further, cardiac resynchronization therapy among patients with wide QRS duration or percutaneous coronary intervention in those with severe coronary artery disease may be beneficial in this same vein.

Patients with severe SMR are often extremely highly morbid, which along with ventricular impairment significantly limits their surgical candidacy. Therefore, multiple trials have been conducted to compare TEER with GDMT in SMR. In the COAPT trial, patients with severe symptomatic SMR and optimal medical treatment were included, with major echocardiographic exclusion criteria similar to the EVEREST II trial.^15^ In COAPT, TEER showed superior significantly improved outcomes in terms of mortality and hospitalization for heart failure compared with GDMT.^16^ In contrast, the MITRA-FR trial that also compared TEER versus GDMT in SMR patients, with similar inclusion and exclusion criteria to the COAPT trial,^17^ showed no difference between both in terms of mortality and heart failure hospitalization.^18^ Recent studies tried to reconcile these two studies by introducing the concept of SMR proportionality, which is determined by the regurgitant orifice area to left ventricular end-diastolic volume ratio.^19^ The higher the ratio, the more disproportionate the SMR is, which also means that the valve, rather than the ventricle, is the main driver of the disease and these patients would benefit more from valve directed therapies.^20^ In fact, Grayburn et al. compared the baseline echocardiographic characteristics of patients included in both trials and found that COAPT patients had disproportionate SMR, which might explain the better outcomes they had with TEER, in contrast to MITRA-FR patients who had proportionate SMR.^19^ Other explanations involve the inclusion of patients without optimized GDMT in MITRA-FR as well as a higher number of patients in MITRA-FR with significant residual MR after clipping.

Atrial dilation and dysfunction, in the absence of ventricular dysfunction, could cause another subtype of SMR, referred to as atrial SMR.^21^ In contrast to changes seen in ventricular SMR, the mitral valve leaflets have shallower tenting angles and height,^22^ which could pose different anatomic challenges to the TEER procedure. In recent retrospective studies, MR reduction to ≤ 1+ was successful in ~80% of atrial SMR cases, which was significantly correlated with lower left atrial volume and higher leaflet to annulus ratio.^23^ When comparing TEER outcomes, patients with atrial SMR demonstrate similar prognosis to those with ventricular SMR, with right ventricular dysfunction and NYHA class IV independently correlated with mortality.^24^

## Preinterventional Echocardiography for Patient Selection

As discussed, optimal imaging prior to TEER procedure is key for successful reduction of MR with minimal adverse outcomes and complications. Acquisition of high-quality imaging of the mitral valve apparatus, including leaflet and coaptation lengths, location of regurgitation jet, and the presence of other anatomical challenges, is necessary for discussion with the interventional cardiologist to determine optimal device size, number, and location.

### Transthoracic Echocardiography

All three trials mentioned above, which assessed outcomes of TEER, included patients with severe chronic MR.^12,16,18^ Severity was assessed on transthoracic echocardiography (TTE) and graded on four stages (Table 1). Stages were defined using three criteria with at least one criterion being quantitative (regurgitant volume, regurgitant fraction, or effective regurgitant orifice area).^25^ Patients needed to have at least a grade 3 MR in order to be considered for TEER. In addition, chronic severe MR can be determined via other parameters. For instance, chronic severe MR is associated with left atrial and ventricular enlargement due to left-sided overload. Severe MR is also classically holosystolic on continuous wave Doppler imaging, whereas mid to late systolic MR tends to be less severe.^26^

**Table 1:** Quantitative and qualitative criteria for grading of mitral regurgitation severity. EROA: effective regurgitant orifice; LVOT: left ventricular outflow tract; PHT: pressure half time


CRITERIA	MILD (1+)	MODERATE (2+)	MODERATE-SEVERE (3+)	SEVERE (4+)

**QUANTITATIVE**				

Regurgitant volume (mL)	< 30	30-40	45-59	≥ 60

Regurgitant fraction (%)	< 30	30-39	40-49	≥ 50

EROA (cm^2^)	< 0.1	0.1-0.2	0.2-0.29	≥ 0.3

**QUALITATIVE**				

Jet width in LVOT	Small in central jets	Intermediate		Large in central jets

Flow convergence	None or very small	Intermediate		Large

Jet density	Incomplete or faint	Dense		Dense

Jet deceleration rate (PHT, msec)	Faint slow, > 500	Medium, 500-200		Steep, < 200

Diastolic flow reversal	Brief, early diastolic	Intermediate		Prominent holodiastolic


### Transesophageal Echocardiography

Due to the proximity of the mitral valve to the esophagus, TEE allows optimal visualization of the mitral valve apparatus (leaflets, chords, and annulus) and surrounding structures (left atrium and pulmonary veins). Morphology of the MR and the MR jet were an essential inclusion criterion used in the trials for ideal procedural outcomes. In the EVEREST II trial, the location of the jet at the A2P2 segment was a major inclusion criterion.^13^ Similarly, in the COAPT trial, a non-commissural location of the jet was a major inclusion criterion.^15^ Other morphological criteria used in these trials are summarized in Table 2.

**Table 2:** Major inclusion and exclusion echocardiographic criteria for transcatheter edge-to-edge repair derived from the COAPT and EVEREST II trials.


MAJOR INCLUSION CRITERIA

The primary regurgitant jet originates from malcoaptiation of the A2 and P2 scallops of the mitral valve (MV)

**MAJOR EXCLUSION CRITERIA**

MV orifice area < 4.0 cm^2^

Width of the flail segment ≥ 15 mm or flail gap ≥ 10 mm

Coaptation depth > 11 mm or vertical coaptation length is < 2 mm

Severe mitral annular calcification

Evidence of calcification in the grasping area of the A2 and/or P2 scallops

Presence of a significant cleft of A2 or P2 scallops

Prior MV surgery or valvuloplasty

Echocardiographic evidence of intracardiac mass, thrombus, or vegetation

History of or active endocarditis or rheumatic heart diseases

History of atrial septal defect or patent foramen ovale associated with clinical symptoms


**Figure 1:**
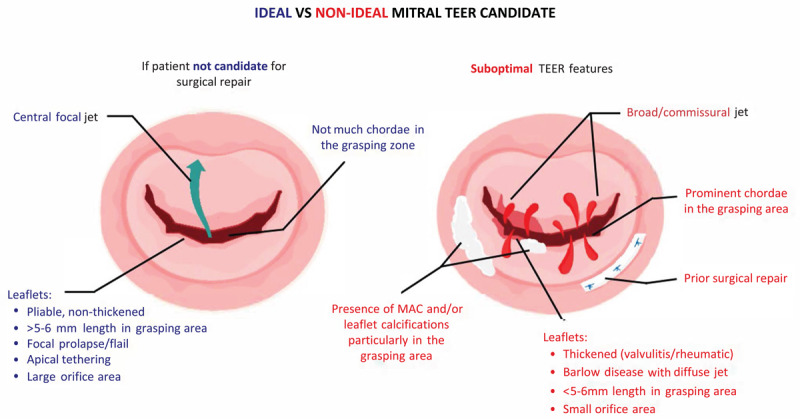
Summary of ideal versus nonideal candidates for transcatheter edge-to-edge repair.

TEE plays a crucial role in assessing the morphology and motion of mitral valve leaflets, such as coaptation length and flail motion. A low coaptation length and a wide flail segment may result in failure of TEER device implantation or procedural complications. Other factors, including the presence of mitral annular calcification (MAC), leaflet thickening due to valvulitis or rheumatic disease, a broad jet, or the presence of chordae in the grasping area, also can lead to suboptimal results (Figure 1). Given its superior temporal resolution, TEE is particularly effective in identifying these factors.

### 3D TEE and Real-time MPR

**Figure 2:**
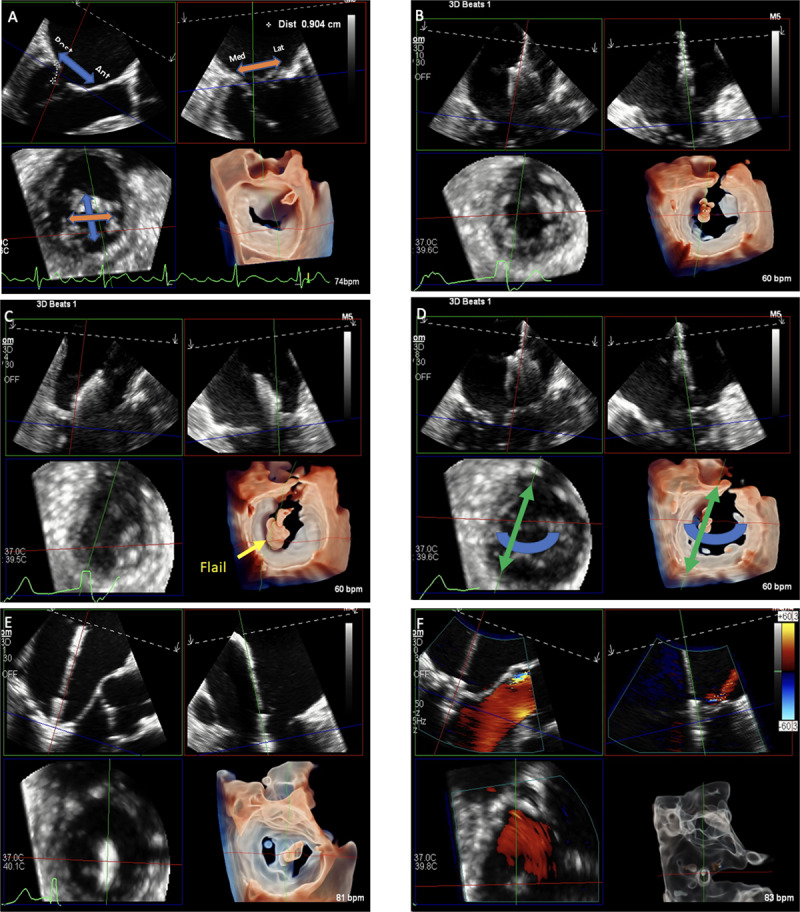
Multiplanar reconstruction (MPR) of the mitral valve using transesophageal echocardiography for assessment and procedural guidance. Multiplanar assessment of the mitral valve **(A)** is done by visualizing the long-axis view (top left plane) of the mitral valve on which anterior and posterior locations could be localized, the bi-commissural (top right) plane where medial and lateral coordinates can be determined; both of these planes will determine the small axis plane (bottom left) and its 3D reconstruction (bottom right). During the procedure, real-time MPR allows **(B)** visualization and adjustment of the device trajectory, **(C)** positioning of the device according to disease location, **(D)** orientation of device for optimal grasping, **(E)** advancement of the device and grasping, and **(F)** evaluation of device attachment and residual disease.

Although 2D TEE offers great visualization, a more detailed assessment is provided using 3D TEE. In the current era, real-time multiplanar reconstruction (MPR) is achieved using commercially available software and allows simultaneous visualization of the mitral valve in three different axis planes (commissural, long axis, and short axis) at the same time. It also allows more accurate localization of the leaflet pathology and resultant regurgitant jet to target during the procedure (Figure 2 A). In addition, it provides a more precise assessment of the regurgitation severity by measuring 3D variables, such as the regurgitant orifice area, which take into account the complex geometry of the regurgitant jet. Finally, 3D MPR allows a detailed assessment of the mitral valve orifice area and annular shape, calcification, and function, which are extremely important in predicting the risk of mitral valve stenosis.

Detailed intraprocedural guidance using real-time MPR (Figure 2 B–F), in addition to the wide range of currently available device sizes, allows extreme precision in positioning and orienting the device, resulting in optimal outcomes without even mild residual disease.

### Intracardiac Echocardiography (ICE)

Intracardiac echocardiography is a potential alternative to TEE in cases where patients are intolerant to TEE. The ICE catheter is advanced to the left atrium after transseptal puncture and placed on top of the mitral valve, allowing good visualization of the valve and multiplanar reconstruction to guide the TEER procedure. Safety and efficacy of ICE in guidance of TEER procedures was reported in several case reports.^27,28^

## Characteristics Preventing Favorable Outcomes Post TEER

**Figure 3:**
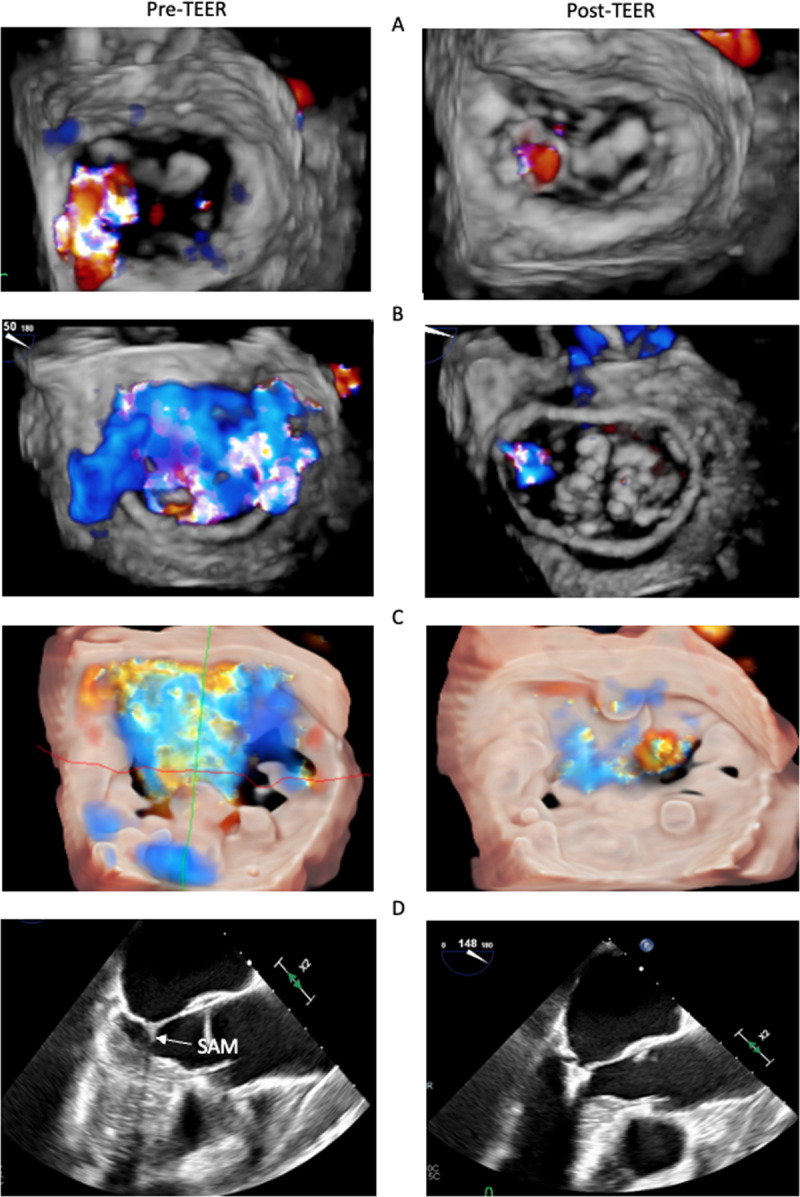
Pre- and post-transcatheter edge-to-edge repair (TEER) echocardiography in challenging cases of TEER in **(A)** commissural mitral regurgitation; **(B)** prior ring annuloplasty; **(C)** severe mitral annular calcification; and **(D)** hypertrophic cardiomyopathy with left ventricular outflow tract obstruction due to systolic anterior motion (SAM).

The most dreaded valve-related adverse outcomes following TEER are mitral stenosis, significant residual MR, and single leaflet device attachment (SLDA). Some key anatomic and clinical characteristics that predict adverse outcomes should be considered before undergoing TEER. Anatomically, thick and stiffened leaflets that may result from valvulitis, chronic rheumatic disease, or radiation heart disease can become even less mobile after plication of a portion of the valve, resulting in a high post-TEER gradient. Mitral annular calcification, in addition to being a risk factor for significant stenosis post TEER, sometimes extends to the leaflets and may increase the risk of leaflet tear.^29^ A retracted or short posterior leaflet could also predispose to insufficient MR reduction. Leaflet perforation or cleft and wide coaptation gap with multiple jets also are to be considered due to high risk for residual MR post TEER. Other anatomical factors not related to the mitral valve can also affect procedural outcomes. For example, delivery of the device and access to the mitral valve could be interrupted by anomalous vena cava anatomy or a history of atrial septal defect corrected using a large device. Clinically, patients with an expected survival of less than 12 months or less than severe MR (despite symptoms) are usually not fit for TEER procedure. Further, the presence of other comorbidities, such as history of chronic obstructive pulmonary disease, advanced-stage renal disease (stage III and IV), and history of atrial fibrillation or flutter, are also associated with worse outcomes in SMR patients undergoing TEER.^30^ Despite these challenges, these cases can be performed (eg, commissural MR, severe MAC, and mitral ring) with adequate pre- and intraprocedural imaging guidance and procedural experience (Figure 3).

Although its incidence is rare, SLDA is a feared procedural complication. SLDA often happens in patients with leaflet calcification, leaflet thickening, or deep leaflet scallops, where grasping both leaflets is challenging; it also is frequently due to poor imaging quality limiting visualization of implantation.^31^ Similar to other complications, preprocedural planning and optimal visualization of the mitral valve apparatus on echocardiography and cardiac CT, in addition to intraprocedural real-time MPR, may help prevent SLDA.

Finally, echocardiography plays a crucial role in determining adverse outcomes and progression of cardiac function following TEER, such as SLDA, recurrence of regurgitation, improvement of pulmonary veins flow, ventricular and atrial remodeling, and strain analysis.^32,33^

## Future Perspectives

**Figure 4:**
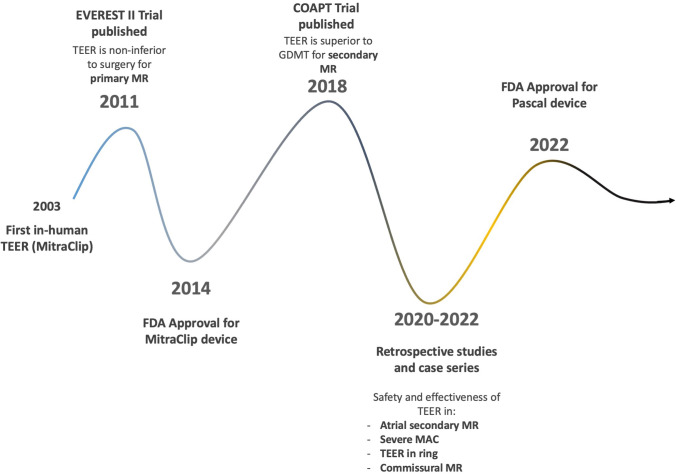
Timeline of scientific and technological advancements of transcatheter edge-to-edge repair since the first in-human procedure. MR: mitral regurgitation; GDMT: goal-directed medical therapy; TEER: transcatheter edge-to-edge repair; MAC: mitral annular calcification

While the newer generation of available devices and procedural advancements has expanded the range of patients currently eligible for TEER (Figure 4), multiple other minimally invasive transcatheter therapies are under investigation to further safely and effectively treat MR patients. For example, the MISCEND trial for the Evoque device (Edwards Lifesciences), the SUMMIT trial for the Tendyne device (Abbott), and the APPOLO trial for the Interpid device (Medtronic) are all ongoing trials for transcatheter mitral valve replacement devices. Another device targeting the mitral valve is the Carillon Mitral Contour System (Cardiac Dimensions), currently under investigation in the EMPOWER trial; it is a newly developed device that simulates the ring annuloplasty intervention by inserting a transcatheter device through the coronary sinus.^34^ None of these devices are FDA approved, although promising preliminary results are emerging.^35,36^ Furthermore, while current guidelines recommend TEER for patients who are not eligible for the surgical procedure, trials such as PRIMARY and REPAIR MR, are currently investigating the differences in clinical outcomes of TEER versus surgical repair in patients with PMR, who are eligible for both surgical and transcatheter interventions.^37,38^

## Conclusion

Mitral valve repair using TEER is a revolutionary minimally invasive therapy for patients with severe MR who are unsuitable for surgical intervention. Adequate patient selection through clinical assessment and advanced imaging modalities are essential to optimize procedural outcomes and prevent adverse events.

## Key Points

- Transcatheter edge-to-edge repair (TEER) is a safe and effective treatment for patients with both primary and secondary mitral regurgitation.
- Although TEER can be associated with certain complications, many of them can be prevented with adequate preprocedural planning and imaging.
- TEER is currently performed in high surgical risk patients; however, ongoing investigations are exploring the feasibility and safety of TEER in moderate surgical risk patients.
